# Lignin phosphorylation to enhance mechanical and physical properties and reduce formaldehyde emissions in plywood panels

**DOI:** 10.1039/d6ra00104a

**Published:** 2026-02-16

**Authors:** Hafida Maarir, Yassine El Khayat driaa, Hassan Charii, Abdelghani Boussetta, Mehdi Mennani, Nabil Grimi, Amine Moubarik

**Affiliations:** a Chemical Processes and Applied Materials Team, Polydisciplinary Faculty, Sultan Moulay Slimane University BP 592 Beni Mellal Morocco Hafida.maarir@usms.ac.ma a.moubarik@usms.ma; b Université de technologie de Compiègne, ESCOM, TIMR (Integrated Transformations of Renewable Matter), Centre de recherche Royallieu CS 60319 – 60203 Compiègne Cedex France; c Laboratory of Chemistry, Biochemistry, Environment, Nutrition and Health Faculty of Medicine and Pharmacy, University Hassan II 20000 Casablanca Morocco; d Higher School of Education and Training, Sultan Moulay Slimane University Beni-Mellal Morocco; e Materials Science, Energy and Nanoengineering (MSN) Department, Mohammed VI Polytechnic University Lot 660, Hay Moulay Rachid 43150 Ben Guerir Morocco

## Abstract

Lignin is gaining more attention for its potential use in adhesives for wood-based composites owing to its accessibility, molecular structure, barrier properties, and potential for chemical modification. In order to assess the physical, thermal, and mechanical performances of alkaline lignin (AL) and its ability to reduce formaldehyde emissions in synthetic phenol formaldehyde (PF) adhesives used to glue plywood panels, AL was surface-modified by a phosphorylation treatment to produce phosphorylated alkali lignin. In this study, lignin was isolated from alkali-treated powdered argan shells and subsequently phosphorylated to obtain phosphorylated lignin (P-AL). The isolated lignin was characterized by two-dimensional (2D-^1^H and ^13^C NMR) heteronuclear single quantum coherence (HSQC) spectra to confirm its successful isolation from argan shells. Both AL and P-AL were then subjected to a series of analytical techniques, including Fourier transform infrared (FTIR) spectroscopy, scanning electron microscopy (SEM) with elemental mapping, X-ray fluorescence (XRF) spectrometry, phosphorus nuclear magnetic resonance (^31^P NMR) spectroscopy, conductimetric titration (to determine the phosphorylation rate), thermogravimetric and derivative thermogravimetric analysis (TGA/DTG) and X-ray diffraction (XRD) analysis. Afterwards, P-AL was formulated with PF resin and applied to plywood panels, following characterizations to evaluate mechanical properties, such as bond strength (BS), modulus of rupture (MOR), modulus of elasticity (MOE), shear strength (SS), and formaldehyde emission (FE). Results showed that lignin phosphorylation had a significantly positive effect on plywood adhesion, especially in terms of FE, which decreased from 2.5 to 1.89 mg^−1^ m^−2^ h^−1^, while maintaining higher mechanical properties, including an MOE of 4144 MPa and an MOR of 66 MPa.

## Abstract 2

Lignin is gaining more attention for its potential use in adhesives for wood-based composites owing to its accessibility, molecular structure, barrier properties, and potential for chemical modification.

## Graphical abstract

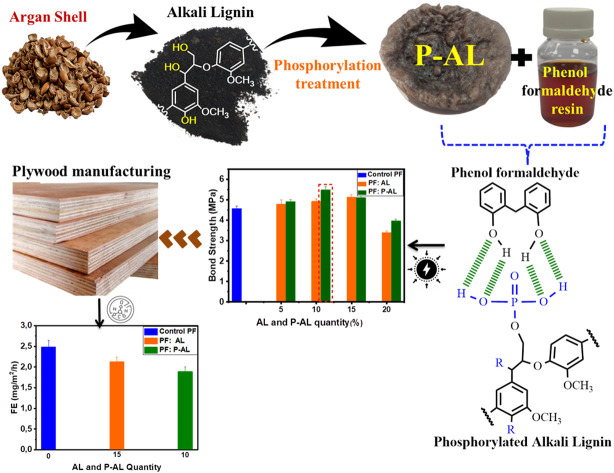

## 1. Introduction

Recently, the search for sustainable resources has been of great importance in order to obtain relevant and environmentally friendly adhesives made up of non-toxic compounds, offering the possibility of manufacturing semi-synthetic or natural adhesives for the successful development of wood composite technology.^1^ Integrating different types of formaldehyde scavengers, such as synthetic, natural (bio-based), and nanoscale components, into conventional synthetic wood adhesives can effectively reduce the emission of harmful free formaldehyde from wood-based panels.^2,3^ Additionally, the surface coating of wood can further contribute to emission reduction.^4,5^ The properties of wood-based products are primarily governed by two key factors: the quality of the wood employed in manufacture and the type of adhesive applied.^6^ Their modification is a common method used to improve the properties of wood-based products. It involves impregnating the veneer with a mixture of urea and potassium carbonate, chemically modifying the adhesives, or adding different types of fillers to enhance the characteristics of veneered plywood composites. Fillers are inorganic or lignocellulosic particles that do not adhere to adhesive formulations, and they are non-volatile and insoluble in these formulations. The goal of modification is commonly to improve the mechanical performance of the materials and thus lower the amount of adhesive used.^7^ Besides, modifying the ingredients of adhesives may give rise to greater reactivity and reduce formaldehyde concentration, which is particularly important in the case of phenol-formaldehyde resins. Lignin is a complex and heterogeneous natural molecule, known to be the second most abundant organic biopolymer on the planet after cellulose. The paper and pulp industry produces approximately 80 million tons of lignin annually, which is a by-product of lignocellulosic biomass.^8^ This natural polymer comprises three different phenylpropane units, namely, H (*p*-hydroxyphenyl), G (guaiacyl) and S (syringyl). The lignins obtained from the industry are technical lignins, such as kraft lignin, ogranosolv lignin, sulphonate lignin, soda lignin, steam explosion lignin and enzymatic hydrolysis lignin.^9–13^ These are by-products generated from various biorefinery processes.^14^ Lignin was poorly developed for industrial applications due to its complex phenolic ring structure, low solubility, and high binding energy, which render it difficult to transform lignin into valuable chemical products. This is why, over the last two decades, lignin has attracted the attention of researchers as a feedstock for the manufacture of value-added products of commercial interest. To this end, chemical functionalization has been studied and is being developed to increase the compatibility of this biopolymer with existing materials.^8^

Lignin, whose composition includes hydroxyl, aromatic, methoxyl and carbonyl groups, can be subjected to functionalization due to its reactive characteristics.^15^ The properties of lignin can be modified through chemical transformations such as esterification,^16^ amination,^17^ sulfonation^18^ and nitration.^19^ The use of chemically modified lignin has gained increasing attention, opening new avenues for the development of new products and paving the way for high-value-added applications. In all these modifications, phosphorylation has attracted specific interest, particularly in the flame-retardant sector.^20–22^ Previous studies have demonstrated the presence of covalent bonds between phosphate groups and the lignin structure and adaptability, involving the reaction of lignin with various phosphorylating agents, including phosphorus pentoxide, phosphoric acid, phosphorus hypochlorite, and ammonium dihydrogen phosphate. These modifications enable improved performance in terms of thermal stability.^23–25^ Besides, lignin phosphate has also been extensively used in corrosion-inhibiting coatings^26^ and as a metal chelating agent.^27^ Lignin is an abundant and inexpensive by-product from the pulp and paper industry, typically costing significantly less than phenol derived from petrochemical resources.^28^ However, the low natural reactivity and heterogeneous structure of lignin often require chemical modifications, such as phosphorylation or other functionalization processes, to improve its adhesive performance, resulting in additional processing costs. Nevertheless, several studies have shown that modified lignin can improve mechanical strength and promote better compatibility within phenolic adhesive matrices, even at low substitution rates.^29^ This partial substitution of fossil-based phenol can thus help offset the costs associated with chemical modifications.^30^ Although certain limitations remain, particularly in terms of performance and industrial scale-up, the optimization of formulations and the development of large-scale processes could promote the commercial viability of lignin-based adhesives, offering both economic and environmental advantages over conventional systems.^29^

On the other hand, there is growing interest in extracting lignin from non-woody biomass such as sugarcane,^31^ flax fiber,^32^ bamboo alfalfa^33^ and wheat straw.^34^ The argan trees (*Argania spinoza*) are the second most common wood species after cedar and oak. It is unique to south-west Morocco and is a member of the Sapotaceae plant family, where argan groves span over 800 000 ha.^35^ Argan trees are known for the highly valued oil extracted from their grains. Argan oil's relevance in Morocco stems from its traditional uses in cooking, healing, and cosmetic products.^36^ The fruit of the argan tree consists of the pulp, nuts and kernels, from which the oil is extracted, representing around 80% of the total fruit weight.^36^ To produce 1 to 2 kg of argan oil, about 6.5 kg of kernels are needed, which are obtained from 60 kg of nuts, requiring roughly 100 kg of fruit in total.

Phosphorylated alkali lignin has not yet been investigated as a filler to enhance mechanical properties and reduce formaldehyde emissions in adhesives for wood-based composites. Phosphorylated lignin in citric acid-sucrose-based resins for cross-linked panels, as reported by Iswanto *et al.*,^37^ is the only reported work that is closely related to our study. They assert that phosphorylated lignosulfonate nanoparticles are integrated into the resin in order to enhance the flame-retardant properties of the panels.

Thus, the purpose of this work was to evaluate waste product recycling *via* the alkali extraction of lignin and its surface modification by phosphorylation to obtain a material with positive effects on the adhesive performance of wood-based composites, specifically the PF adhesives used in plywood. The morphology, chemical functionalities, and thermal properties of phosphorylated lignin were evaluated, along with the mechanical properties and formaldehyde emissions of plywood panels.

## 2. Materials and methods

### 2.1. Materials

Argan shells, a lignocellulosic raw material, were used in this study. They were collected from the Agadir region (southern Morocco). The composition of the material is approximately 42% cellulose, 20% hemicellulose, and 34% lignin. All analytical grade chemicals used for lignin isolation, including sodium hydroxide (NaOH) and sulfuric acid (H_2_SO_4_), were purchased from Sigma-Aldrich (France) and used without further purification. The following products were used for the phosphorylation process: diammonium hydrogen phosphate ((NH_4_)_2_HPO_4_) and urea ((NH_2_)_2_CO). For conductometric titration, sodium hydroxide (NaOH, >97%), sodium chlorite (NaClO_2_), and hydrochloric acid (HCl) were used. All chemicals were purchased from Sigma-Aldrich and used without further modification.

### 2.2. Methods

#### 2.2.1. Alkaline isolation of lignin from argan shells

**Fig. 1:**
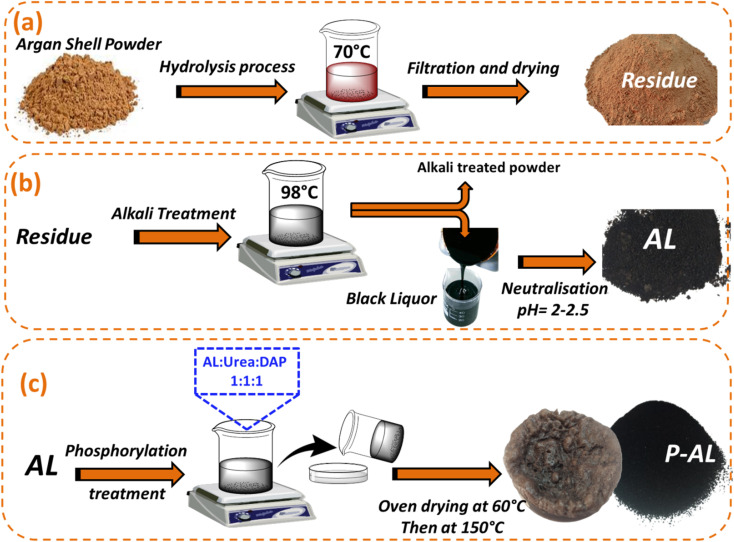
Schematic illustration of (a) the hydrolysis of argan shells (AS), (b) the alkali delignification process, and (c) the phosphorylation of alkali lignin (AL).

The lignin isolation process from the AS material was carried out using the alkaline process according to the same procedure as reported.^38^ The AS powder was initially treated with distilled water at 70 °C for 2 hours under mechanical stirring (Fig. 1(a)) at a solid-to-liquid ratio of 1 : 10 (w : v) in order to remove water-soluble impurities. After the initial treatment, the material was filtered to separate the water-soluble fractions. The remaining residue was then subjected to an alkaline treatment using an aqueous sodium hydroxide (NaOH) solution for 1 hour and 30 minutes at a temperature ranging from 90 to 98 °C, under mechanical agitation, as shown in Fig. 1(b). The alkali-treated AS was then subjected to efficient filtration to separate the black liquor from the cellulose fibers. Lignin was recovered by adding drops of sulfuric acid, which precipitated the acidified lignin. The precipitate was washed several times with distilled water for partial purification and then air-dried to obtain the final lignin.

#### 2.2.2. Phosphorylation of isolated argan lignin

Fig. 1(c) illustrates the simplified process leading to phosphorylated lignin (P-AL). In this process, 1 g of di-ammonium hydrogen phosphate (DAP) and 1 g of urea catalyst were mixed with distilled water, followed by the addition of 1 g of lignin powder under mechanical stirring at room temperature for 20 minutes, as described in a previous work.^39^ The mixture was oven-dried at 60 °C overnight for dehydration. Following this, to start the phosphorylation reaction, the resulting dehydrated material was heated to 150 °C for 1 hour. Finally, the obtained phosphorylated lignin was filtered, washed and dried.

## 3. Characterizations and analysis

Two-dimensional (2D) heteronuclear single-quantum coherence (^1^H and ^13^C HSQC) NMR analysis was performed for an isolated lignin sample using a Bruker Avance 400 MHz spectrometer. Approximately 60 mg of each sample was dissolved in 1.0 mL of DMSO-d_6_. The ^13^C NMR spectra were recorded at 25 °C with a total of 30 000 scans using standard Bruker pulse sequences optimized for heteronuclear correlation experiments. The acquired data were processed and analyzed using TopSpin 4.3.0 software, enabling precise peak assignment and structural interpretation. The resulting spectra provided critical insights into the molecular structure and interaction dynamics of the lignin sample.

The chemical structure of the samples was studied using Fourier transform infrared spectroscopy with a JASCO FT/IR 4600 type A instrument. Spectra were recorded in the 4000–400 cm^−1^ region with an accumulation of 16 scans and a resolution of 4 cm^−1^. This analysis was performed in order to evaluate the functional groups present in both materials, AL and P-AL.

The effect of chemical treatments on argan powder morphology, starting with hydrolysis, followed by alkaline treatment and ending with phosphorylation, was analyzed using scanning electron microscopy (SEM, Zeiss Evo 10). The surface of the sample was coated with a thin layer of conductive gold using an ion sputtering apparatus.

The phosphorus content (P) in both untreated alkali lignin (AL) and phosphorylated alkali lignin (P-AL) samples was measured using an X-ray fluorescence spectrometer (XRF). This technique enables rapid, direct detection, providing accurate quantitative values within seconds.

^31^P NMR spectroscopy was performed in solution using a 400 MHz Bruker spectrometer with Topspin software. The modified lignin (0.06 g) was dissolved in 1 ml of DMSO-d_6_ and transferred to NMR tubes. ^1^H NMR spectra were recorded with 64 scans, while ^31^P NMR spectra were recorded with 61 scans.

According to Ait Benhammou *et al.*,^40,41^ the phosphorylation rate of lignin extracted from argan shells was determined using a modified conductimetric titration technique. To begin the titration, 5 ml of NaCl (0.01 M) solution was mixed with 100 ml of distilled water, and then 0.3 g of the P-AL sample was added to the mixture under mechanical stirring for 10 minutes. The pH was adjusted to 2–2.5 using hydrochloric acid (0.1 M). Subsequently, NaOH (0.1 M) was added gradually until the pH reached 11. The charge content was calculated using the following equation:
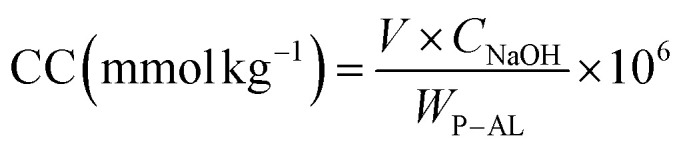
where *C*_NaOH_ is the concentration of NaOH (0.1 M), *V*_NaOH_ is the added volume (*L*) and *W*_P–AL_ is the weight of the phosphorylated alkali lignin sample (*g*).

The thermal stability and degradation behavior of the samples were evaluated using thermogravimetric analysis (TGA) and derivative thermogravimetry (DTG) with a LABSYS Evo TGA 1600 instrument (SETARAM). About 20 mg of each sample was pyrolyzed at temperatures ranging from 25 °C to 700 °C with a heating rate of 10 °C min^−1^ under an air atmosphere to prevent oxidation effects.

The numerous phase types in the examined AR, AL and P-AL materials were characterized using an X-Ray diffractometer (D2 PHASER, BRUKER). Cu Kα radiation (*k* = 1.54056 Å) was used to scan the powdered materials over a 2*θ* range of 5° to 40°. The current and voltage were set to 40 mA and 40 kV, respectively.

## 4. Formulation and application of adhesives

### 4.1. Adhesive formulation

The preparation of unmodified and phosphorylated alkali lignin/PF resins was as per earlier reports.^42,43^ A PF resol resin with a solid content of 46 wt% (viscosity of roughly 450 cP) was prepared by combining formaldehyde and phenol in a 2.2 : 1 ratio using sodium hydroxide (7.3 wt%) as a catalyst. The resol resin was prepared in a 2 L glass reactor with mechanical stirring and temperature control. Adequate amounts of reagents were introduced to the reactor according to the established formulation. The reaction was monitored at 90 °C, and the viscosity of the resulting resol was measured at 25 °C. The formed resins were prepared by copolymerizing AL and P-AL with PF, respectively, in a gel form at weight ratios (w/w) of 5 : 95, 10 : 90, 15 : 85 and 20 : 80.

#### 4.1.1 Resins bond strength

To determine the stiffness and strength of the resin bonds, resins with varying lignin contents (5, 10, 15, and 20 wt%) were tested using an Instron M500-50 AT testing machine at a speed of 1 mm min^−1^. The maritime pine (*Pinus pinaster*) veneers were cut into rectangular pieces measuring 2.5 cm × 11.5 cm × 3 mm. Resins were applied to a 2.5 cm × 2.5 cm area at one side of one end of the pieces. The adhesive dispersion rate was 120–130 g m^−2^ dry weight, as reported in previous studies.^44^

### 4.2. Plywood manufacturing and testing

Plywood is a composite panel produced from thin pieces of wood veneer. In this case, five-layer plywood panels (250 mm × 250 mm × 10 mm) were made with resins in the middle and two 4% moisture-content Maritime pine (*Pinus pinaster*) veneers on the top and bottom. Plywood bonded with AL : PF and P-AL : PF resins was hot-pressed at 12 bar and 160 °C for 6 minutes. Fixed bonding settings were used to simulate industrial plywood manufacturing processes. To achieve a complete reaction, a six-minute press duration was used. Each resin was represented by five laboratory-scale plywood panels. The mechanical properties of plywood panels, which are widely utilized in many applications, were then investigated. The dry tensile strength, modulus of rupture, and modulus of elasticity were measured according to EN 314 (1993)^45^ and EN 310 (1993)^46^ standards, respectively.

### 4.3. Formaldehyde emission

Formaldehyde emissions (FE) from plywood panels were obtained in accordance with the European standard ISO/CD 12460-4 using a glass desiccator with a volume of 10 L. Three samples (15 × 5 × 1 cm^3^) were placed inside the desiccator for the test. The formaldehyde released over 24 hours from all the samples, at room temperature and 45% relative humidity, was subsequently absorbed into a Petri dish containing 30 mL of distilled water placed inside the desiccator. The photometric method was used to determine the amount released. Each resin formulation was tested in triplicate to ensure reproducibility.

## 5. Results and discussion

### 5.1. Nuclear magnetic resonance (^1^H NMR) spectroscopy

**Fig. 2:**
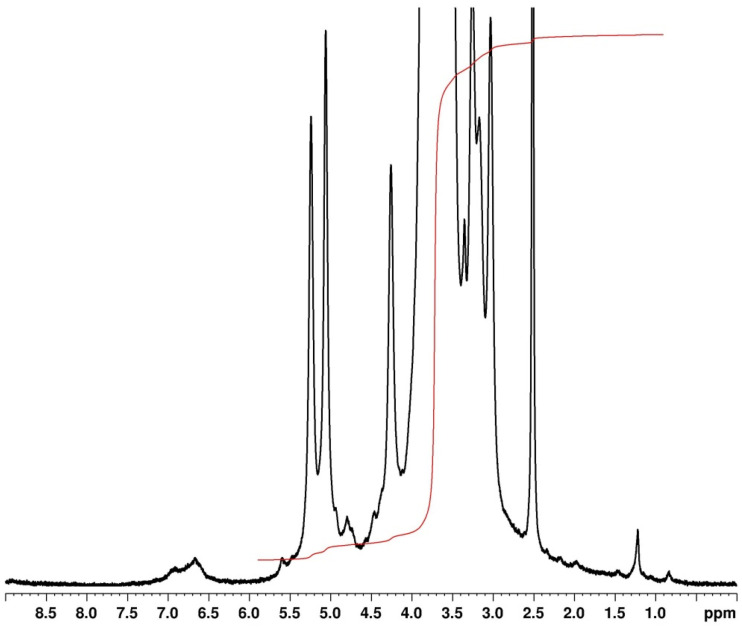
Proton NMR analysis of lignin.

The chemical structure of argan lignin was investigated by proton nuclear magnetic resonance (^1^H NMR) spectroscopy using DMSO-d_6_ as the solvent. Fig. 2 presents the ^1^H NMR spectrum of the extracted lignin sample, which correlated with other studies.^47,48^ Signals observed in the range of 6.5–7.8 ppm are assigned to the aromatic and vinyl protons of syringyl (S) and guaiacyl (G) units. A large signal appearing between 4.9 and 5.5 ppm corresponds to the aliphatic protons associated with lignin side chains (Hα protons of β-O-4′ structures). Sharp resonances detected in the 4–4.3 and 4.7 ppm regions are attributed to methoxyl protons, characteristic of both S and G units and β-O-4 linkages, respectively. The signals around 3–3.4 ppm are attributed to aliphatic hydroxyl groups. The residual water and DMSO-d_6_ solvent are identified by distinct signals at 3.46 ppm and 2.50 ppm, respectively. Additionally, a small resonance at 1.20 ppm corresponds to the methylene protons of the aliphatic chains (Table 1).

**Table 1:** ^1^H–^13^C NMR signal assignments of structural units in alkali extracted lignin

Region	Signal (*δ*_C_/*δ*_H_)	Assignment	Structural description
Aromatic – S units	103–107/6.6–7.2	S_2/6_	Protons on C2/6 of syringyl units
Aromatic – G units	110–115/6.8–7.2	G_2_	Proton on C2 of guaiacyl units
	114–118/6.6–6.9	G_5_	Proton on C5 of guaiacyl units
	119–125/6.7–7.4	G_6_	Proton on C6 of guaiacyl units
Methoxyl groups	55–57/4–4.3	O–CH_3_	Methoxy substituents on S and G units
β-O-4 linkages	70–75/4.7–5.0	Cα–Hα	Benzylic carbon attached to oxygen (α-alcohol)
	82–86/4.1–4.5	Cβ–Hβ	β-Carbon involved in the β-O-4 ether linkage
	59–63/3.2–3.8	Cγ–Hγ	Primary γ-alcohol in β-O-4 substructures
Aliphatic hydroxyl groups	70–80/4.0–4.8	Cα–OH	Benzylic alcohols formed during alkaline treatment
Carbonyl groups (oxidized units)	190–200/—	Cα = O	Trace oxidized lignin structures (often weak in HSQC)
Solvent	39–40/2.5	DMSO-d_6_	Residual solvent signal

### 5.2. 2D HSQC NMR

**Fig. 3:**
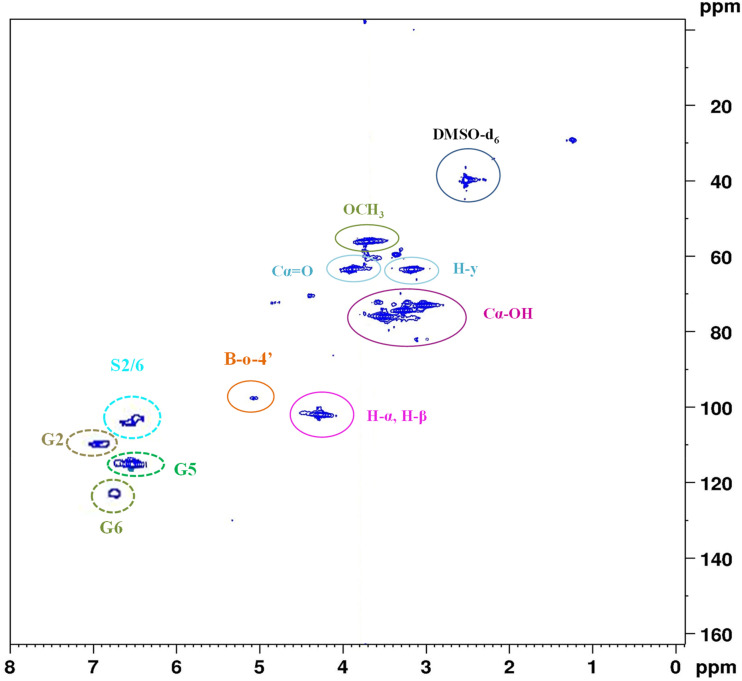
2D HSQC NMR spectra of alkali lignin (AL).

The structural characteristics of alkaline lignin were also evaluated using 2D HSQC (^1^H–^13^C) NMR spectroscopy in a DMSO-d_6_ solution, as shown in Fig. 3. The analysis clearly demonstrates the presence of syringyl (S) and guaiacyl (G) structures, which are prevalent in alkaline lignin. The presence of these structures indicates that the sample was not significantly compromised during processing. In the aliphatic region, the correlations related to the β-O-4 inter-unit bridges remain evident, indicating that the ether bonds in the alkaline lignin structure are partially preserved. The observation of signals corresponding to the α, β, and γ positions in the side chain indicates the presence of the pendant aryl ether bonds. Furthermore, the presence of signals due to benzylic alcohol moieties indicates lignin decomposition and/or the formation of hydroxyl groups during the alkaline treatments. The presence of methoxyl groups indicates the prevalence of substituted aromatic rings, predominantly syringyl (S) and guaiacyl (G) units. Therefore, HSQC spectral analysis indicates that alkaline lignin retains a complex aromatic structure, with readily available aliphatic and phenolic hydroxyl groups. This observation clearly demonstrates the modification and the preservation of lignin molecules during the alkaline isolation process.

### 5.3. ATR/FT-IR analysis

**Fig. 4:**
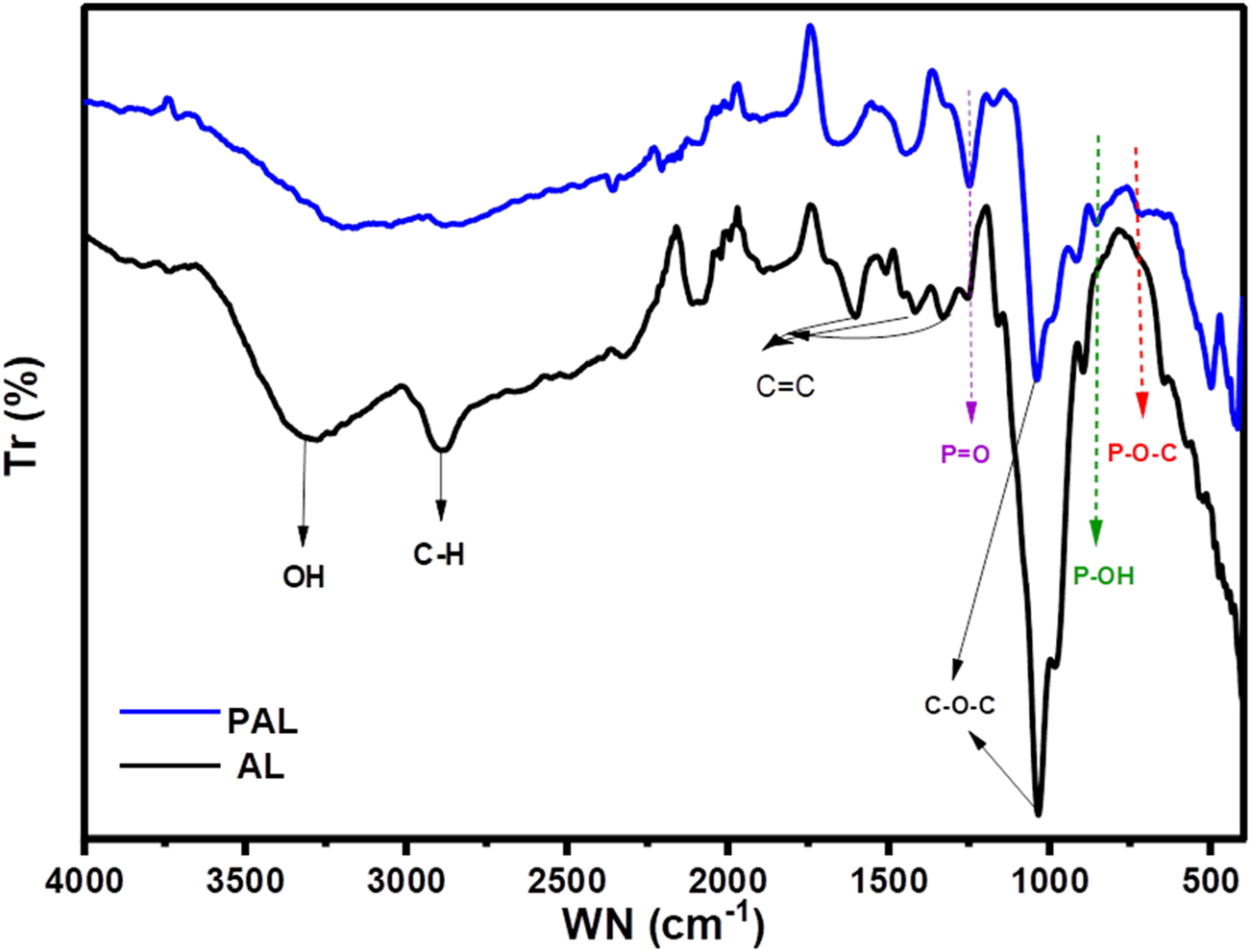
FTIR spectra of alkali lignin (AL) and phosphorylated alkali lignin (P-AL).

In the ATR-FTIR spectra of AL and the modified lignin P-AL, shown in Fig. 4, the band at 3343 cm^−1^ corresponds to the O–H stretching vibration,^37^ whereas the bands at 2918 cm^−1^ and 2865 cm^−1^ are attributed to the C–H stretching vibrations of methylene groups.^49^ The weak band at 1726 cm^−1^ is attributed to the stretching vibrations of carbonyl groups present in the esters.^50^ The weak band at 1224 cm^−1^ and the strong band at 1020 cm^−1^ correspond to C–O stretching vibrations in ester groups.^37^ The bands appearing at 1265 cm^−1^ and 855 cm^−1^ are specific to the guaiacyl (G) units of lignin.^23^ In addition, new peaks such as 910 cm^−1^ are attributed to the presence of P–OH groups, a peak at 1236 cm^−1^ represents the P=O groups,^51,52^ and the band at 715 cm^−1^ is assigned to the P–O–C linkages. The appearance of the latter is due to the disappearance of the O–H stretching band at 3343 cm^−1^. These changes provided strong evidence that the lignin molecules were successfully modified by grafting phosphate groups.

### 5.4. Scanning electron microscopy (SEM) and element mapping

**Fig. 5:**
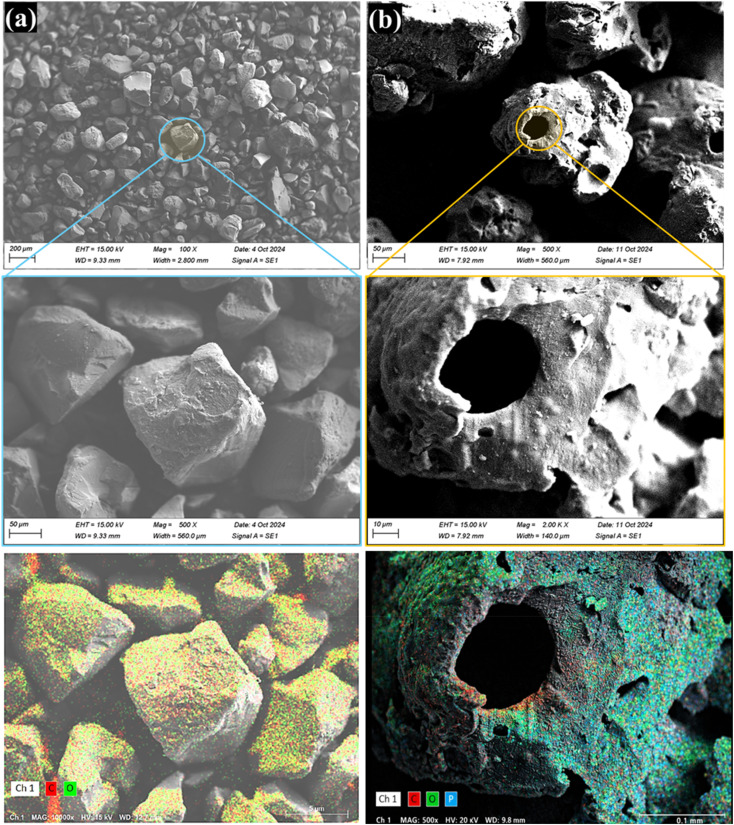
SEM images and elemental mapping of alkali lignin (a) and phosphorylated alkali, lignin (b).

SEM was used to visualize the surface morphology of the particles (Fig. 5). The images of all samples are presented, highlighting important morphological changes in AL after phosphorylation. The P-AL sample exhibits a more porous structure, with voids and pores, as well as sharper edges.^53^ These porous structures are pressed together with larger particles compared to those in alkaline lignin, indicating the effect of the phosphorylation process, which leads to particle size distribution and agglomeration.^20^ As shown in Fig. 5, the elements C, O, and P are depicted on the surface of the phosphorylated sample.^54^ Phosphorus is uniformly distributed across the entire surface of the phosphorylated lignin particles, with a higher relative density compared to oxygen and carbon. This distribution confirms the successful phosphorylation treatment, highlighting the incorporation of phosphorus into the lignin structure.

### 5.5. Phosphor nuclear magnetic resonance ^31^P NMR

**Fig. 6:**
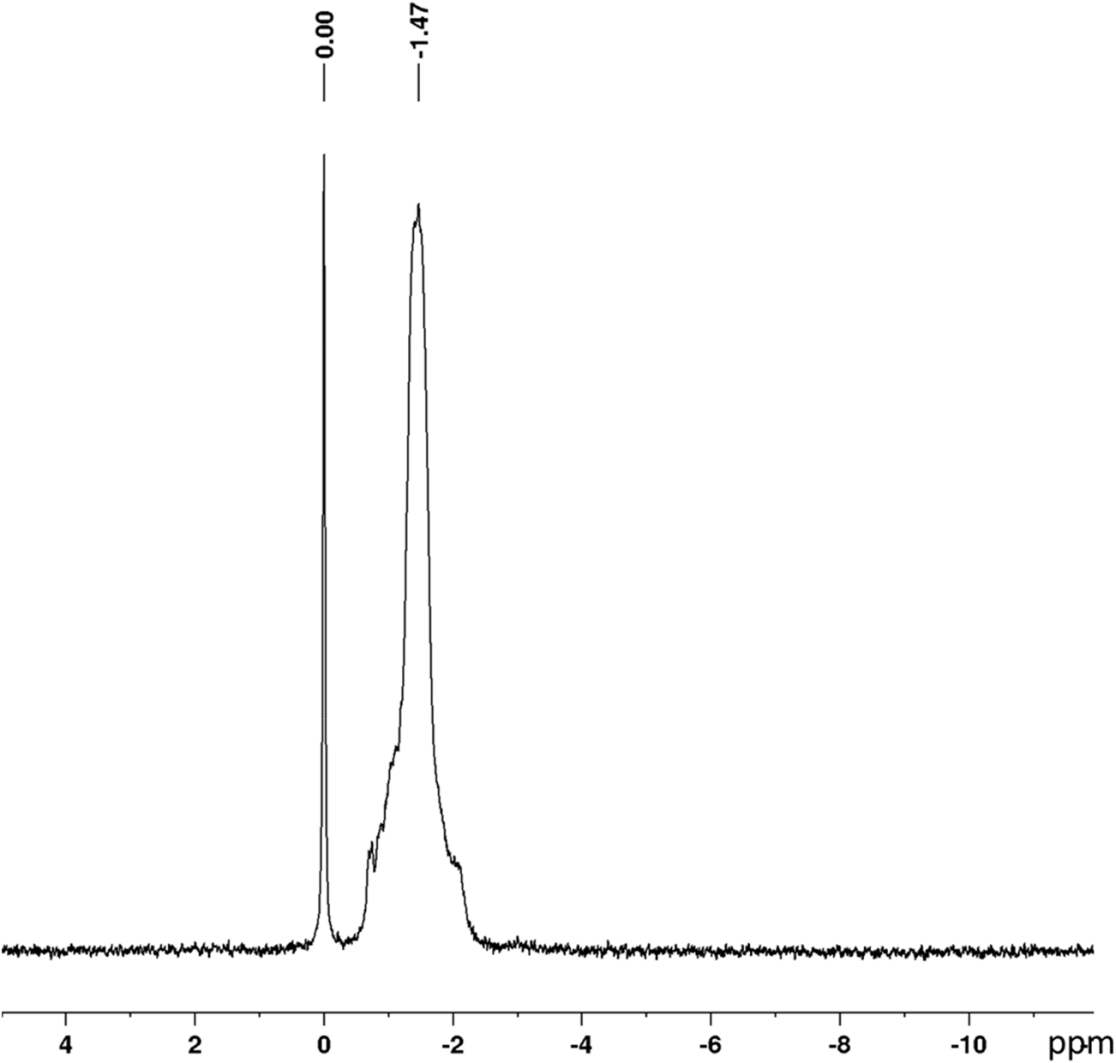
Phosphorus nuclear magnetic resonance (^31^P NMR) of phosphorylated alkali lignin.

The ^31^P NMR analysis (Fig. 6) revealed phosphorus resonance at −1.47 ppm. According to Prieur *et al.*,^20^ this chemical shift corresponds to hydroxyl groups on the aliphatic side chains, which appear in the ^1^H NMR spectrum at 3.78 and 4 ppm. This signal can therefore be attributed to the P–O–R′ phosphate groups, where *R*′ corresponds to aryl fragments of the lignin structure.^55^ These results confirm that alkaline lignin was successfully phosphorylated, with the phosphate groups covalently bonded to the phenolic and aliphatic hydroxyl functionalities. Furthermore, the presence of a signal at approximately 0 ppm indicates residual and unreacted phosphoric agents.

### 5.6. X-ray fluorescence spectrometer (XRF)

**Fig. 7:**
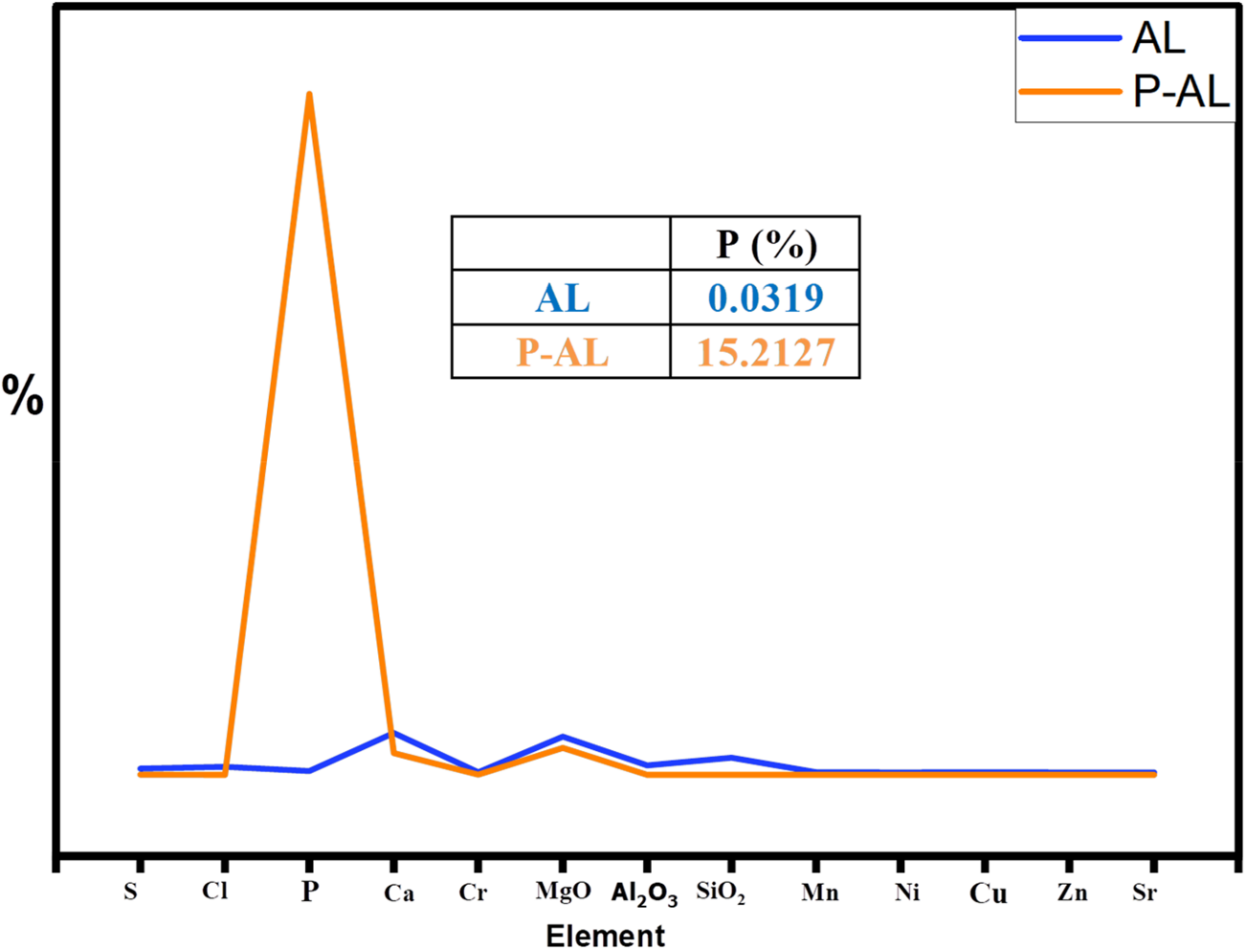
X-ray fluorescence (XRF) analysis of alkali lignin (AL) and phosphorylated alkali lignin (P-AL).

In the present study, XRF measurements were used to assess the elemental content of unmodified alkali lignin (AL) and phosphorylated alkali lignin (PAL), with a particular focus on phosphorus (P). The resulting dataset is presented in Table 2 and Fig. 7. The results indicate a minor presence of phosphorus (P) in unmodified lignin (AL), which is consistent with the absence of a phosphorus source during the material formation. In contrast, following the phosphorylation process, the phosphorus content of the modified lignin (P-AL) was approximately 15%, thereby indicating that the phosphorylation reaction was successful. This substantial increase in the P signal suggests that the phosphorus groups were effectively incorporated into the lignin structure. The percentage obtained by XRF is directly proportional to the total amount of phosphorus in the sample.^56^

**Table 2:** Elemental composition of alkali lignin (AL) and phosphorylated alkali lignin (P-AL), obtained by XRF analysis

Element	S	Cl	P	Ca	Cr	MgO	Al_2_O_3_	SiO_2_	Mn	Ni	Cu	Zn	Sr
AL (%)	0.0863	0.1263	0.0319	0.8802	0.0021	0.8022	0.1563	0.3253	0.0047	0.0007	0.002	0.0012	0.001
P-AL (%)	0.0061	—	15.2127	0.4852	0.0020	0.6033	—	—	—	0.0004	0.001	0.0009	0.0006

### 5.7. Determination of phosphorylation rate

**Fig. 8:**
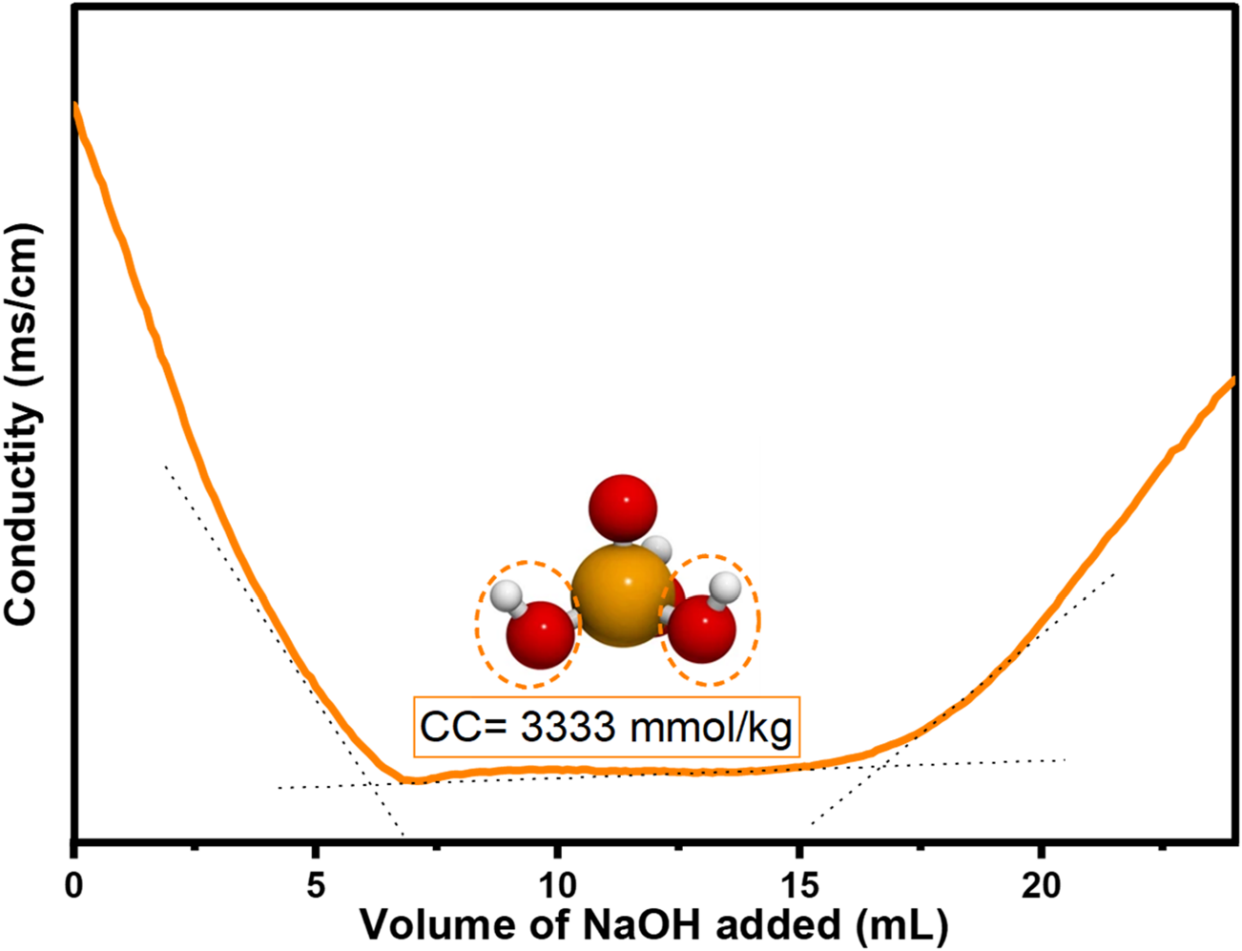
Conductimetric titration curve of the phosphorylated alkali lignin sample.

The results of the conductometric titration used to determine the phosphorylation rate are displayed in the titration curve in Fig. 8. The conductance of phosphorylated lignin changes with the addition of NaOH, forming three distinct regions: the first region shows the consumption of H^+^ ions. The second region shows a plateau, which indicates neutralization between ions, allowing the calculation of the amount of charge of the phosphate groups. The last region indicates an excess of OH^−^ ions from the NaOH solution.^57^ The results obtained show a high filler content, reaching a maximum of 3333 mmol kg^−1^ in phosphorylated lignin. This suggests that the sample is very rich in hydroxyl groups from the phosphoric reagent, confirming the successful completion of the alkali lignin phosphorylation process.

### 5.8. TGA/DTG analysis

**Fig. 9:**
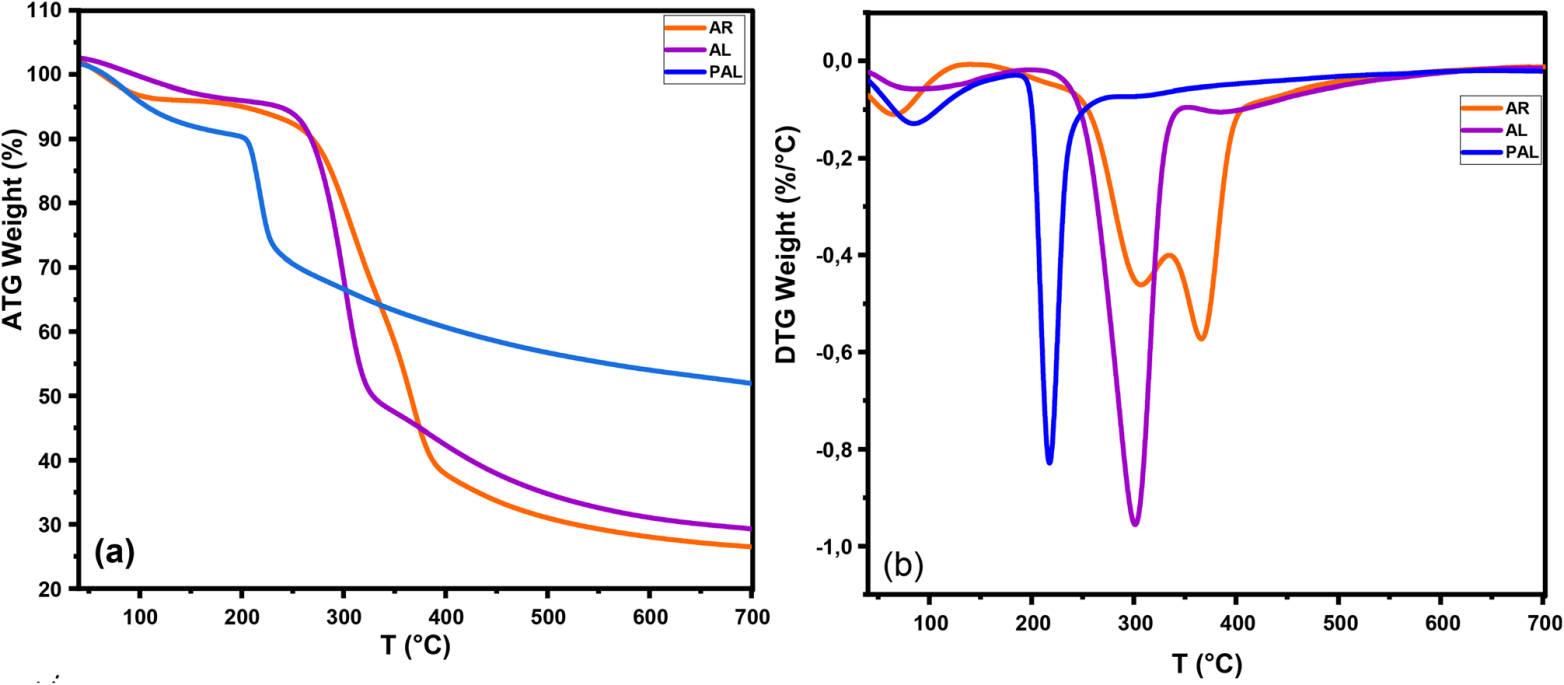
TGA and DTG curves of argan raw (AR), unmodified alkali lignin (AL), and phosphorylated alkali lignin (P-AL).

Thermal analysis evaluates the percentage of weight loss of a material when subjected to heating in a controlled atmosphere.^37^ Weight loss in different temperature ranges gives indications of material composition, including inert deposits and volatile substances, as well as thermal stability. As shown in Fig. 9, the material's mass loss can be divided into three areas: evaporation, degradation and carbonization. In the course of the evaporation phase, all AR, AL, P-AL samples exhibited mass losses of approximately 3%, 4%, and 6%, respectively. This initial weight loss is attributed to the vaporization of low-molecular-weight volatile compounds. At high temperatures, material degradation induces the decomposition of the propanoid chain within the lignin polymer, resulting in the release of lighter molecules, such as CO_2_ and CO. Concurrently, this process facilitates the formation of phosphorus-activated water molecules.^58^ During the degradation stage, the onset temperatures of the AR (325.00 °C) and AL (300.00 °C) samples were higher than that of the P-AL (224.71 °C), indicating that phosphorylation reduces the thermal stability of lignin. However, the weight loss of the AR sample at this stage (60%) is higher than that of the AL sample (50%), and even higher than that of the P-AL sample (25%). At 700 °C, the remaining weights of the AR, AL and P-AL samples were approximately 25%, 30% and 55% respectively. These results indicate that phosphorylation enhances the char-forming ability of lignin, contributing to improved thermal resistance. The P-AL sample shows excellent thermal stability compared to unmodified lignin. Consequently, the decomposition of P–O–C groups results in the formation of a charcoal layer that effectively inhibits further penetration of gases and heat, thereby reducing thermal degradation.^59,60^

### 5.9. X-ray diffraction analysis

**Fig. 10:**
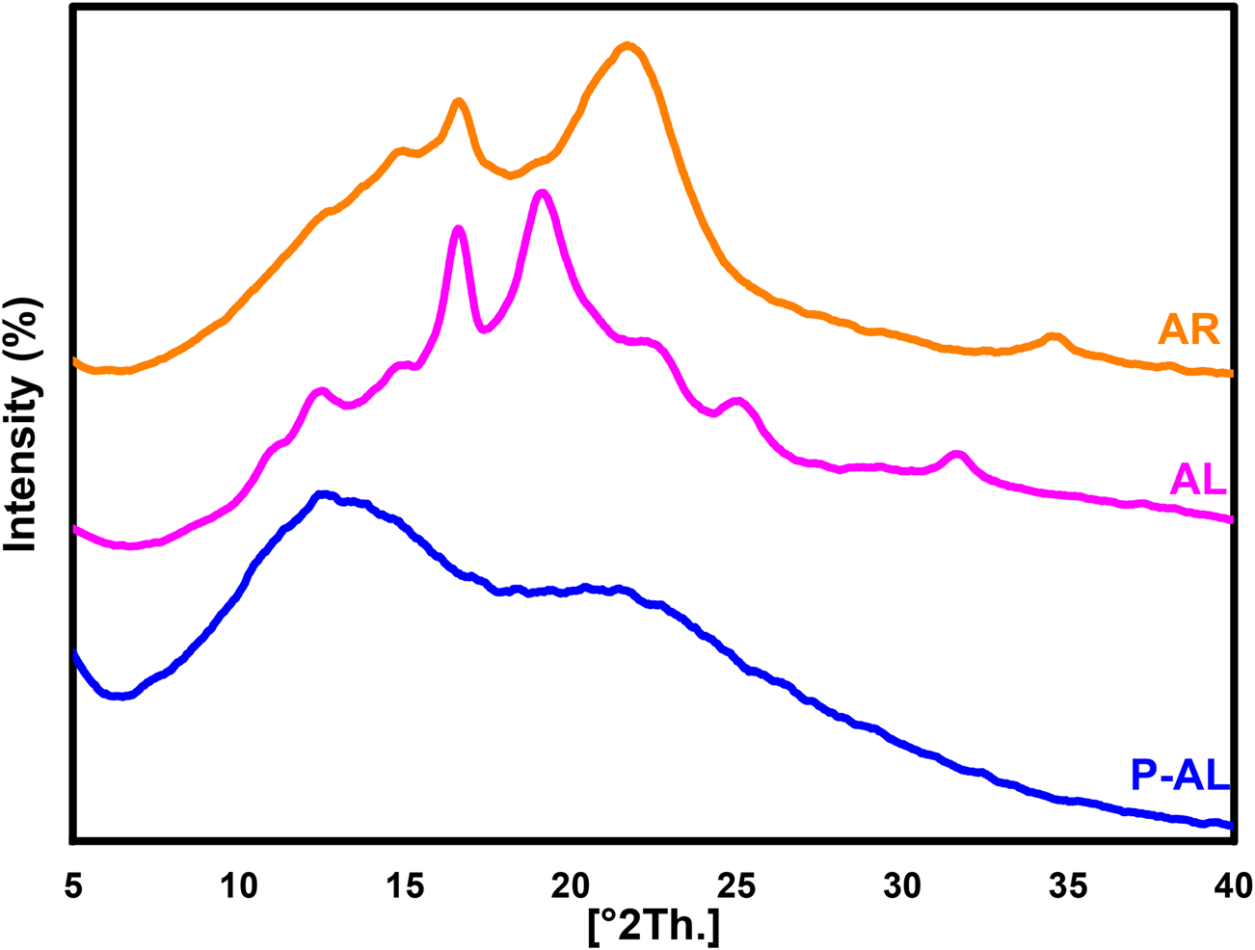
X-ray diffraction patterns of argan raw (AR), alkali lignin (AL) and phosphorylated alkali lignin (P-AL).

The diffractogram presented in Fig. 10 shows the X-ray diffraction (XRD) patterns of argan raw (AR), alkali lignin (AL), and phosphorylated alkali lignin (P-AL). The crystalline structure of cellulose is revealed by the first spectrum, corresponding to AR, which shows a characteristic peak at 22° and does not appear in the spectrum of the alkali-treated lignin samples, indicating that cellulose was effectively separated from lignin during the delignification process. The peaks at 16.8° and 19° observed in the AL spectrum are related to the lignin's structure. The decrease in their intensity in the P-AL spectrum indicates that phosphorylation changed the lignin structure by adding phosphoric groups and enhanced the transition towards a more amorphous character.

### 5.10. Proposed chemical structure of phosphorylated lignin

**Fig. 11:**
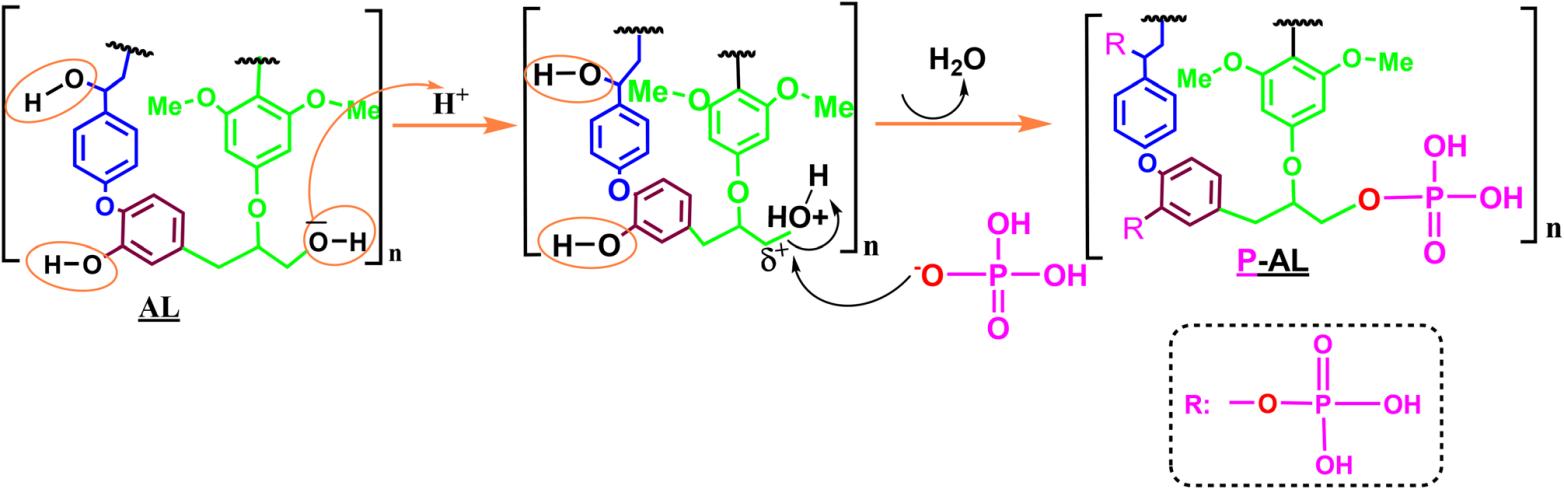
Proposed chemical structure of phosphorylated lignin.

The grafted phosphate groups are considered the polycondensation site for the subsequent generation of polyphosphate ester linkages. Additionally, the most common functional groups in lignin are hydroxyl groups, comprising both phenolic and aliphatic (–OH) groups.^61^ These hydroxyl groups play an essential role in various chemical reactions, such as phosphorylation.^62^ Lignin is generally phosphorylated by a nucleophilic substitution mechanism, in which hydroxyl groups are replaced by phosphate groups. This process often uses diammonium phosphate (DAP), which is activated by urea under heating. Heating DAP in the presence of urea leads to its decomposition and the formation of reactive phosphate species, principally, the dihydrogen phosphate anion (H_2_PO_4_^−^). This electron-rich anion acts as a nucleophile and can attack the electrophilic carbon atoms to which the hydroxyl groups are linked in the lignin structure.^63^Fig. 11 shows a possible phosphorylation pathway mechanism during the reaction and under the reaction conditions.

### 5.11. Adhesive properties

The pH, solids content, gel time at 120 °C and viscosity of PF, AL//PF, and P-AL//PF adhesives were measured at varying amount ratios and are presented in Table 3. The pH levels of all adhesives were practically identical. The solid content increased with filler quantity in both AL//PF and P-AL//PF adhesives, reaching 61% and 63%, respectively, at a weight ratio of (20 : 80; w/w), compared to the control PF adhesive (45%).

**Table 3:** Physical properties of plywood samples prepared with PF, AL//PF and P-AL//PF adhesives

Adhesives ratio (%)		pH	Solid content, %	Gel time at 120 °C, s	Viscosity, cP
Control PF	0//100	11.17	45	984	425
AL//PF	5//95	11.19	49	809	491
	10//90	11.18	53	771	532
	15//85	11.16	56	768	582
	20//80	11.15	61	724	746
P-AL//PF	5//95	11.18	48	811	496
	10//90	11.17	54	783	557
	15//85	11.16	57	756	626
	20//80	11.16	63	718	755

Similarly, viscosity increased with the level of substitution, peaking at a weight ratio of (20 : 80; w/w) for the AL//PF (746 cP) and P-AL : PF (755 cP) adhesives compared to the control PF adhesive (425 cP). This increase can be attributed to the presence of stiff particles in the resin formulation. The addition of lignin and phosphorylated lignin into PF resins acts as a matrix-reinforcing agent, increasing the density of the formulation. The glue thus develops a viscous consistency, allowing the formulation to be tailored to the specific requirements of wood composites.^64^

In addition, adhesives prepared with AL and P-AL at all weight ratios had the quickest gel times compared to the control PF adhesive. In particular, lignin and phosphorylated lignin can modify the chemical reactivity of the system by activating reactive sites with phosphate groups, thus promoting faster cross-linking. Furthermore, the characteristics of the AL//PF and P-AL//PF adhesives did not surpass the Chinese requirements for wood adhesives (GB/T 14732) ^41^, indicating that they are appropriate and adaptable materials for practical applications.

### 5.12. Bonding quality of formulated adhesives in plywood and formaldehyde emissions

**Fig. 12:**
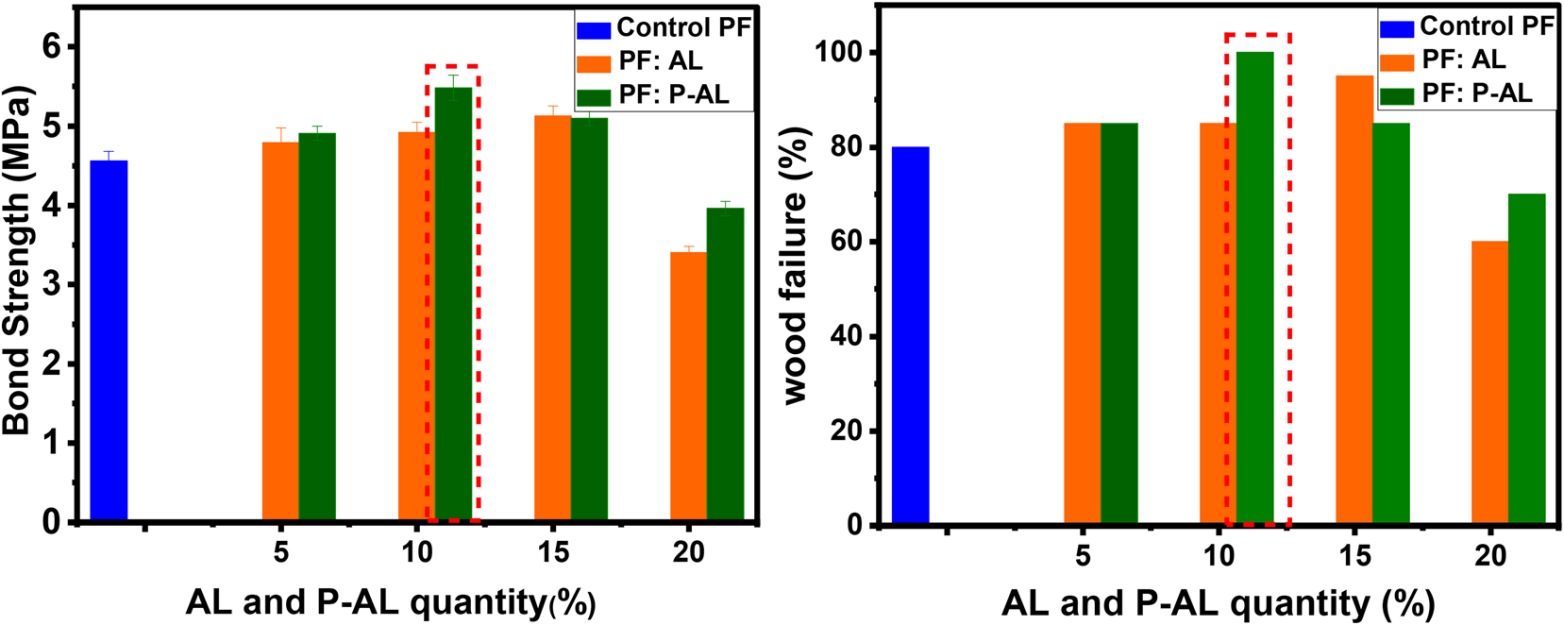
Bond strength and wood failure performance of adhesive formulations in the manufactured plywoods.

The ability of the wood modification to affect the bonding interface between the adhesive and wood plies largely depends on the quality of the wood itself. Bond strength and the percentage of wood failure are the typically used indicators of bonding quality. As shown in Fig. 12, various percentages of the PF resin, unmodified (AL) and modified (P-AL) fillers ranging from 0, 5, 10, 15 and 20% were evaluated. The bond strength was determined as a function of the deformation of plywood core layers. The results show that the untreated control material exhibited a bond strength of 4.56 MPa with a wood failure value of 80%. Upon the addition of AL and P-AL, the bond strength increased gradually across all filler ratios. Optimal values were achieved at 15% AL (5.13 MPa with 95% wood failure) and 10% P-AL (5.48 MPa with 100% wood failure). However, at a filler content of 20%, the bond strength began to decline. These results demonstrate that the modified plywood is stronger compared to plywood panels bonded only with the PF control resin. This improvement is attributed to the increased number of reactive sites in the materials created by the extraction process, followed by phosphorylation, which results in a more cross-linked and rigid polymeric network within the plywood structure.

**Fig. 13:**
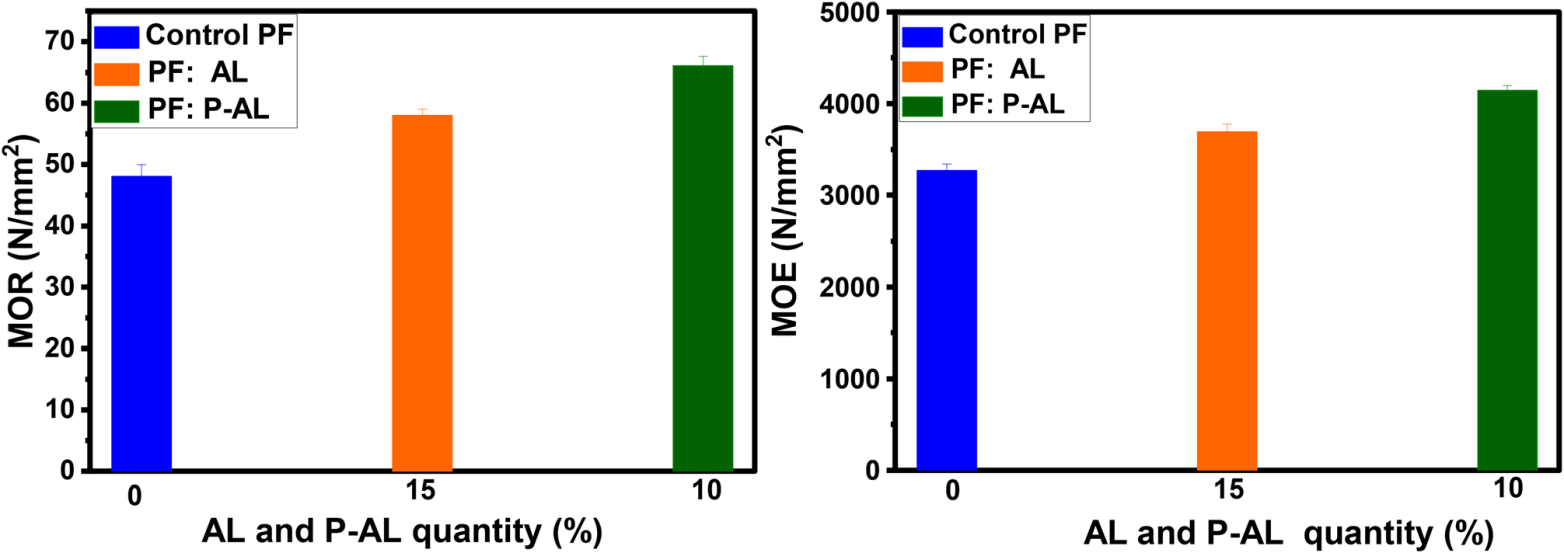
Mechanical properties of manufactured plywood: modulus of rupture (MOR) and modulus of elasticity (MOE) for different adhesive formulations.

Particles of alkali lignin (AL) and phosphorylated alkaline lignin (P-AL) were evaluated as fillers in the formulation of a phenol formaldehyde-based adhesive at different weight ratios (5, 10, 15, and 20% of the solid content) to assess their ability as partial substitutes for the PF resin and enhance the tensile properties of plywood panels. To achieve this, the plywood bonded with the optimal AL : PF (15 : 85; w/w) and P-AL : PF (10 : 90; w/w) adhesives were examined for their mechanical properties and compared to plywood bonded with the control PF resin (0 : 100; w/w). Fig. 13 summarizes the data collected for the dry modulus of rupture (MOR), dry modulus of elasticity (MOE), and shear strength (dry, cold, and boiling water). The wood composite bonded with the PF resin (0 : 100; w/w) exhibited an MOR of 48 N mm^−2^ and an MOE of 3267 N mm^−2^. Composites bonded with the optimal AL : PF (15 : 85; w/w) adhesive showed a significant improvement, with an MOR of 58 N mm^−2^ and an MOE of 3692 N mm^−2^. Similarly, the P-AL : PF (10 : 90; w/w) resin showed an increase in MOR and MOE of 66 N mm^−2^ and 4144 N mm^−2^, respectively, compared to the control PF resin. This phenomenon is attributed to the hard phenolic particles, which whether unmodified or chemically modified, improve the elasticity and fracture toughness of wood composites compared to the unfilled resin. At microscopic levels, the wood substrate is rough, porous, and have fissures. The mechanical interconnecting theory might thus be used to explain the effects of adhesive penetration into the wood structure, its rigidity as a key in wood surfaces, and the establishment of a strong link between the adhesive and the wood surface.^43^ In addition, phosphorylated lignin plays a key role in enhancing the strength of PF adhesives owing to its high concentration of hydroxyl groups resulting from phosphorylation. These hydroxyl groups facilitate the formation of effective hydrogen bonds with the hydroxyl groups present in the PF resin network. These interactions between molecules promote harmony between lignin and the phenolic matrix, reduce the mobility of the polymer chains, and contribute to the development of a denser and more coherent adhesive structure. Furthermore, enhanced interactions at the adhesive–wood interface facilitate improved stress transfer, resulting in increased bonding strength, elasticity, and fracture toughness in the resulting wood composites. This demonstrates that adhesives containing lignin particles could penetrate wood and solidify with the wood substrate once heated.

**Fig. 14:**
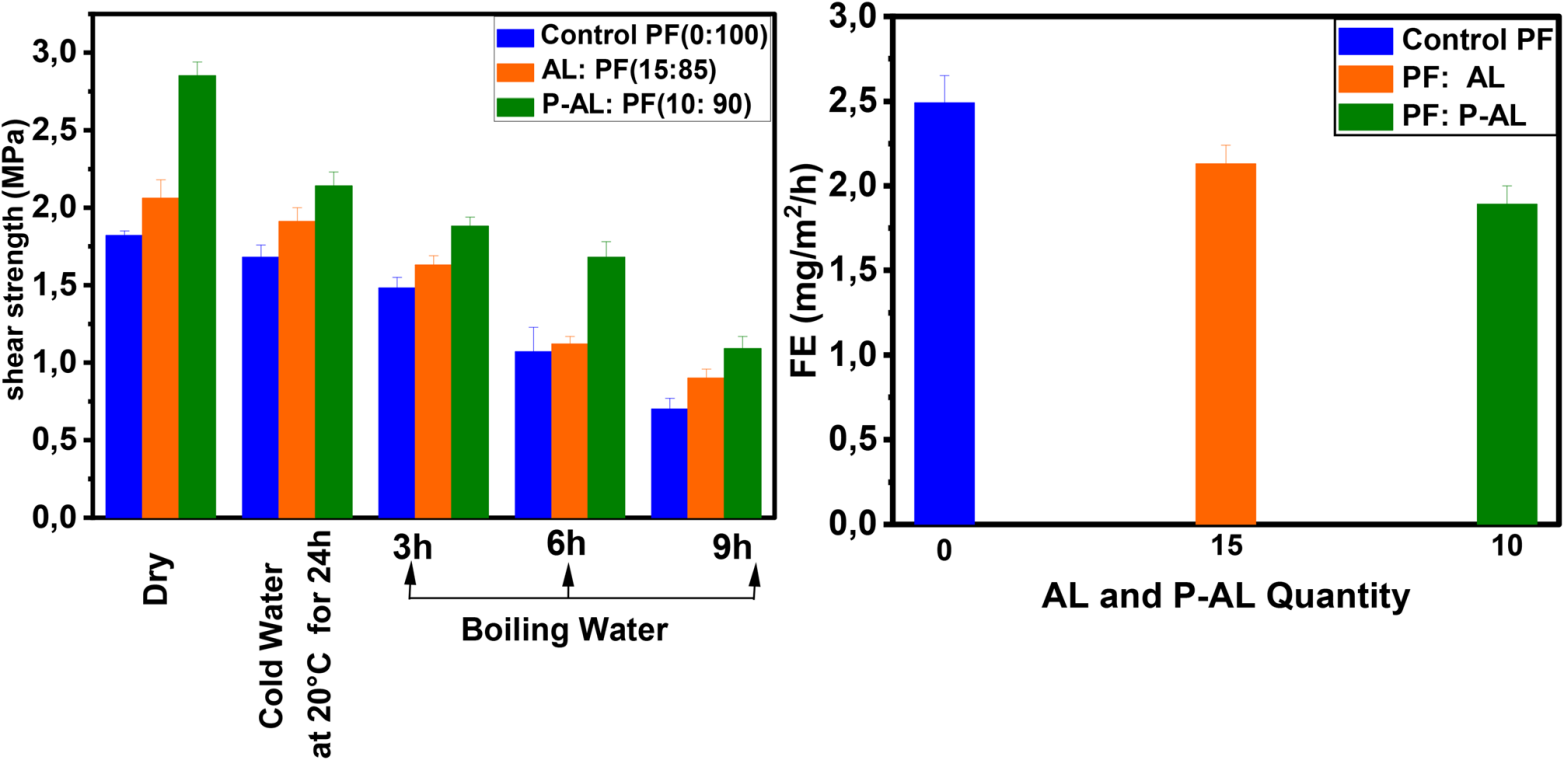
(a) Shear strength (SS) and (b) formaldehyde emission (FE) of plywood.

Table 4 and Fig. 14(a) show the shear strength (SS) of five plywood composites bonded with three different adhesives: the control PF resin, AL : PF (15 : 85; w/w), and P-AL : PF (10 : 90; w/w). The shear strength of the PF control resin (0 : 100; w/w) under dry, cold water, and boiling water (9 hours) conditions were 1.82 N mm^−2^, 1.68 N mm^−2^ and 0.7 N mm^−2^, respectively. The shear strength values obtained for the optimal AL/PF (15 : 85 w/w) adhesive were higher than those of the control PF resin, reaching 2.06 N mm^−2^ (+13.18%), 1.91 N mm^−2^ (+13.7%), and 0.90 N mm^−2^ (+28.57%) under dry, cold-water, and boiling-water conditions, respectively. While for P-AL : PF (10 : 90; w/w), the resin performed better in the dry, cold water, and 9 hour boiling tests, reaching 2.85 N mm^−2^ (+56.59%), 2.14 N mm^−2^ (+27.38%), and 1.09 N mm^−2^ (+55.7%), respectively, compared to the PF control resin. Interestingly, the P-AL : PF resin (10 : 90 w/w) demonstrated much superior mechanical performance than the AL : PF (15 : 85; w/w) resin, which could be attributed to the presence of phosphate groups from the phosphorylation treatment. The results demonstrate that the shear strength levels differ between the dry and boiling water tests. The shear strength is lowered, mostly due to heat effects, which may cause the resin to lose tenacity and hence reduce its capacity to create strong bonds. Water adsorption, on the other hand, causes wood fibers to deteriorate, resulting in dilatation.

**Table 4:** Shear strength (SS) of plywoods under dry, cold, and boiling water conditions

Adhesives formulation (w/w)	Dry	Cold water at 20 °C for 24 h	Boiling water (h)		
			3	6	9
Control PF (0 : 100)	1.82	1.68	1.48	1.07	0.7
AL//PF 15 : 85	2.06	1.91	1.63	1.12	0.9
P-AL//PF 10 : 90	2.85	2.14	1.88	1.68	1.09

As shown in Fig. 14 (b), wood composites prepared with the AL : PF (15 : 85; w/w) resin exhibited reduced formaldehyde emissions of 2.13 mg ^−1^m^−2^ h^−1^ compared to the control PF resin (2.49 mg ^−1^m^−2^ h^−1^). The P-AL : PF (10 : 90; w/w) formulation reduced formaldehyde emissions to 1.89 mg ^−1^m^−2^ h^−1^. The use of natural lignin particles (AL and P-AL) and PF penetration into the wood structure contributed to the reduction of formaldehyde emissions from plywood panels. These findings are consistent with those reported by Moubarik *et al.*^42^ The higher formaldehyde emission of panels bonded with the control PF resin can be attributed to the greater number of bridges in the PF resin. This is a result of a part of free formaldehyde accessible in the PF resin that does not react with phenol during synthesis but can instead react with the active sites of modified lignin during the LPF resin synthesis, causing lower formaldehyde emissions in plywood panels.^65^

## 6. Conclusions

Phosphorylated alkali lignin (P-AL) extracted from argan shells was successfully incorporated into phenol-formaldehyde (PF) resins and was fully characterized through chemical and thermal analyses. Covalent bonds were formed between the phosphate groups and lignin, which improved its chemical structure and significantly increased its thermal stability compared to unmodified lignin. An increase in viscosity, gel time, and solid content was observed when the PF resin was partially replaced with P-AL at 20%. The P-AL : PF (10 : 90) formulation yielded the most optimal results in plywood panel production. An increase of up to 37.5% in MOR and up to 26.8% in MOE was observed, accompanied by an increased shear strength and a 24.1% reduction in formaldehyde emissions.

These results prove that phosphorylation not only optimizes the functional characteristics of lignin but also promotes the development of highly efficient, low-emission bio-based wood composites. This research highlights a sustainable chemical approach that utilizes underutilized residues of argan shells, thereby offering a renewable alternative to petroleum-based phenol and opening the door to environmentally friendly applications in the wood industry.

## Conflicts of interest

There are no conflicts to declare.

## Data Availability

The data of this study are available from the corresponding author upon reasonable request.
